# Assessing the rigidity of thermoplastic masks for head immobilization effectiveness in stereotactic radiosurgery

**DOI:** 10.1002/acm2.70058

**Published:** 2025-02-20

**Authors:** Iris Pasion Apale, Adam Agnew, Daniel Foley

**Affiliations:** ^1^ University College Dublin Belfield Dublin Ireland

**Keywords:** Brainlab thermoplastic mask, ExacTrac SGS, head immobilization, motion management, rotational displacement, stereotactic radiosurgery (SRS)

## Abstract

This study compared three Brainlab thermoplastic masks—Cranial 4pi basic, stereotactic (Close Mask V2), and open‐face—to see how well they limited head movement during Stereotactic Radiosurgery (SRS). Using a head phantom, we tested rotational movements (pitch, yaw, and roll) and measured displacements with the ExacTrac system. The open‐face mask had the smallest mean displacements (pitch: 0.14 ± 0.03°, yaw: 0.11 ± 0.02°, roll: 0.16 ± 0.03°) and performed slightly better than the stereotactic mask in pitch (0.20 ± 0.04°, *p* = 0.0173). The stereotactic mask performed similarly in yaw (0.09 ± 0.02°) and roll (0.16 ± 0.04°). The basic mask showed much more movement (pitch: 0.44 ± 0.13°, yaw: 0.28 ± 0.07°, roll: 0.26 ± 0.07°), making it less suitable for SRS. These results apply to the solid two‐piece masks tested here and show that both the open‐face and stereotactic masks provide reliable immobilization for accurate SRS treatments.

## 1 INTRODUCTION

In radiotherapy, particularly for the treatment of head‐and‐neck cancers, the precision of patient positioning is crucial. This becomes even more critical in Stereotactic Radiosurgery (SRS), a high‐precision technique designed to deliver concentrated radiation doses to small targets. A minor misalignment in patient positioning can result in suboptimal tumor targeting, increasing the risk of unnecessary radiation exposure to surrounding healthy tissues, potential side effects, and even a geometric miss of the tumor, which may lead to disease progression.^1^, ^2^

Thermoplastic masks are commonly used for patient immobilization to ensure accurate and reproducible positioning throughout treatment.^3^, ^4^, ^5^ These masks aim to restrict patient movement and improve the precision of radiation delivery. However, despite their widespread use, there are ongoing questions about the effectiveness of different mask designs, particularly in reducing head movement during SRS.^6^, ^7^, ^8^

This study evaluates the performance of three thermoplastic masks developed by Brainlab for use with their ExacTrac system: the Cranial 4pi basic mask (Close Mask V1), Cranial 4pi stereotactic mask (Close Mask V2), and Cranial 4pi open‐face mask (Open Mask). The focus is on quantifying the masks' ability to restrict rotational head movements in pitch, yaw, and roll—movements that can significantly affect the precision of SRS.^9^, ^10^, ^11^ A head phantom was used to simulate these movements, with forces incrementally applied to assess each mask's rigidity and effectiveness in limiting displacement. The results aim to provide radiotherapy team members with insights into the comparative performance of these masks, helping them choose the most suitable immobilization device for enhancing the accuracy of SRS treatments.

## 2 METHODS

### 2.1 Equipment and thermoplastic mask preparation

**FIGURE 1:**
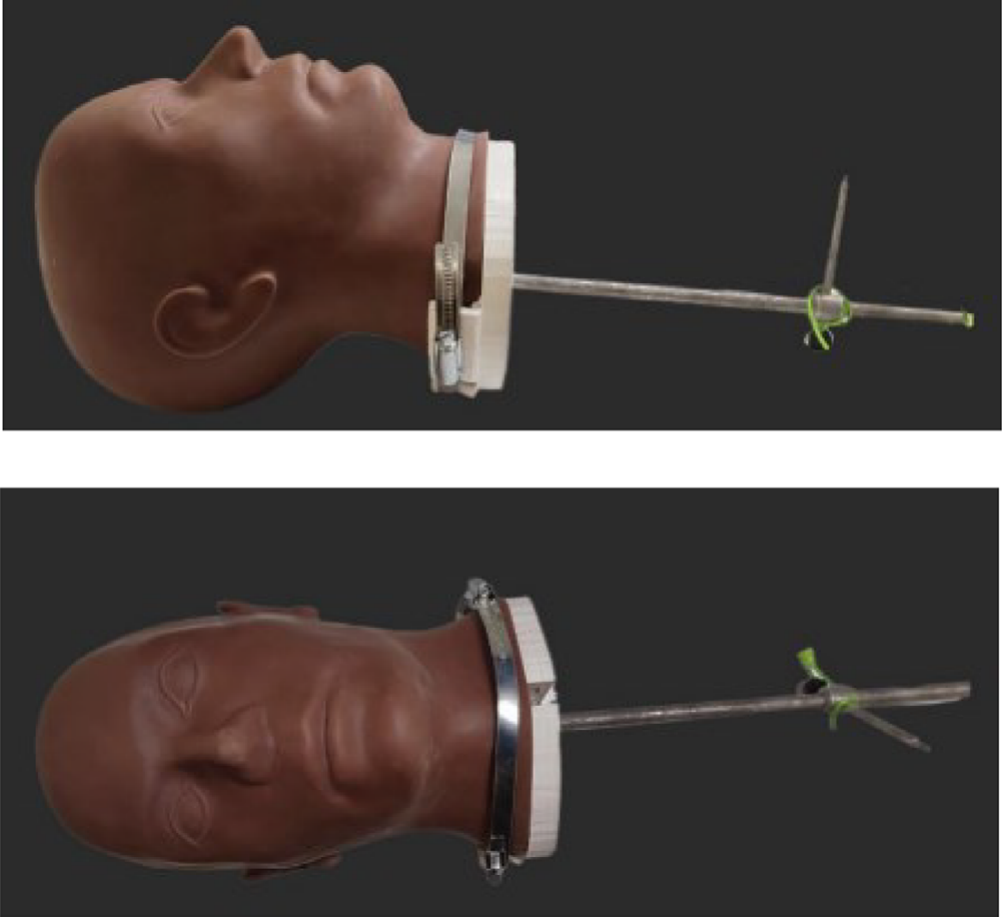
Side view (top) and front view (bottom) of head phantom with central rod and 3D‐printed neck clamp.Metal brace around neck to keep 3D‐print neck clamp secure.

An ExacTrac head phantom (Brainlab AG, Munich, Germany) was used to simulate patient head movement. The phantom is designed with a central rod (30 cm length, 1 cm diameter) and a custom 3D‐printed neck clamp (Figure 1) allowing for the attachment of weights to simulate head motion in different directions.

**FIGURE 2:**
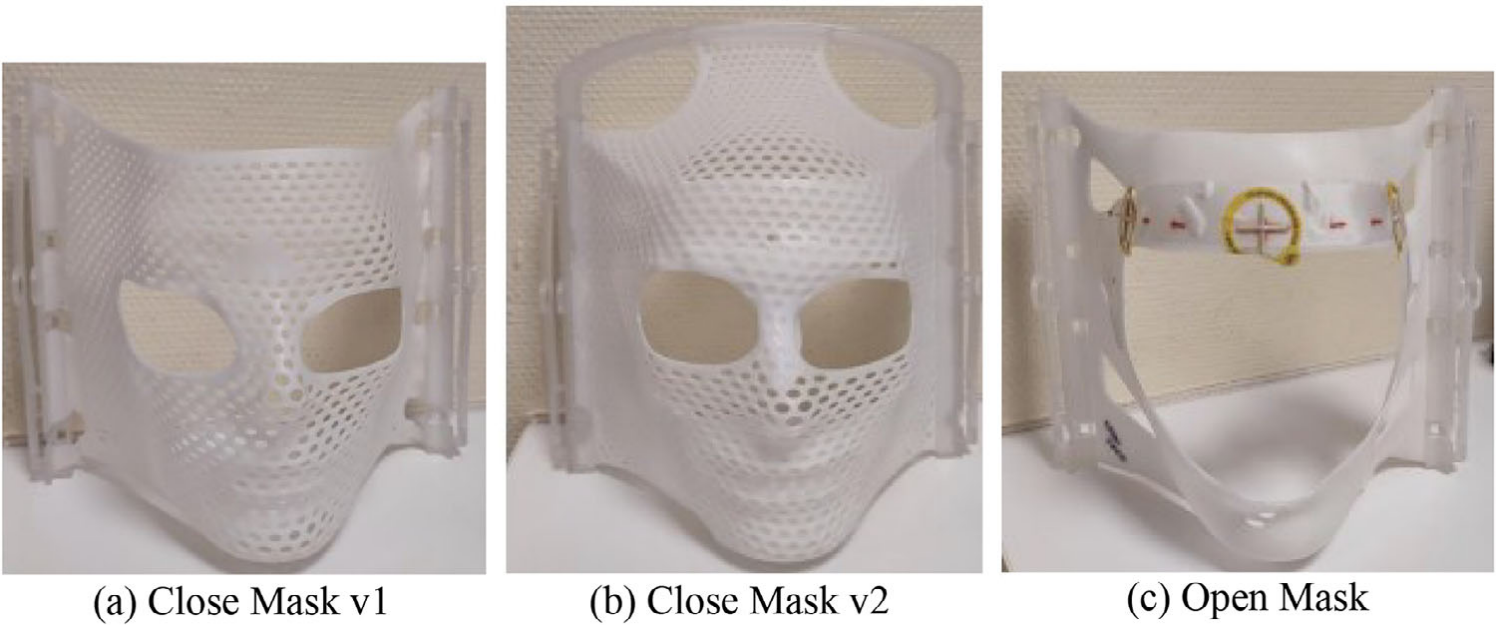
Brainlab thermoplastic masks.

The study used three types of Brainlab thermoplastic masks: the Cranial 4pi basic mask, the Cranial 4pi stereotactic mask, and the Cranial 4pi open‐face mask (Figure 2). Each mask was immersed in a water bath at 70°C for 10 min before being molded onto the phantom's head. Once fitted, the masks were allowed to cool and set.

### 2.2 Phantom alignment and ROI marking

The head phantom, fitted with each mask, was carefully positioned to align with the isocentre of the linac. The 3D optical surface of the phantom's face was matched with the reference contour taken from the treatment planning CT. To track movements, a Region of Interest (ROI) was marked on the 3D‐printed face of the phantom, representing the Planning Target Volume (PTV).

**FIGURE 3:**
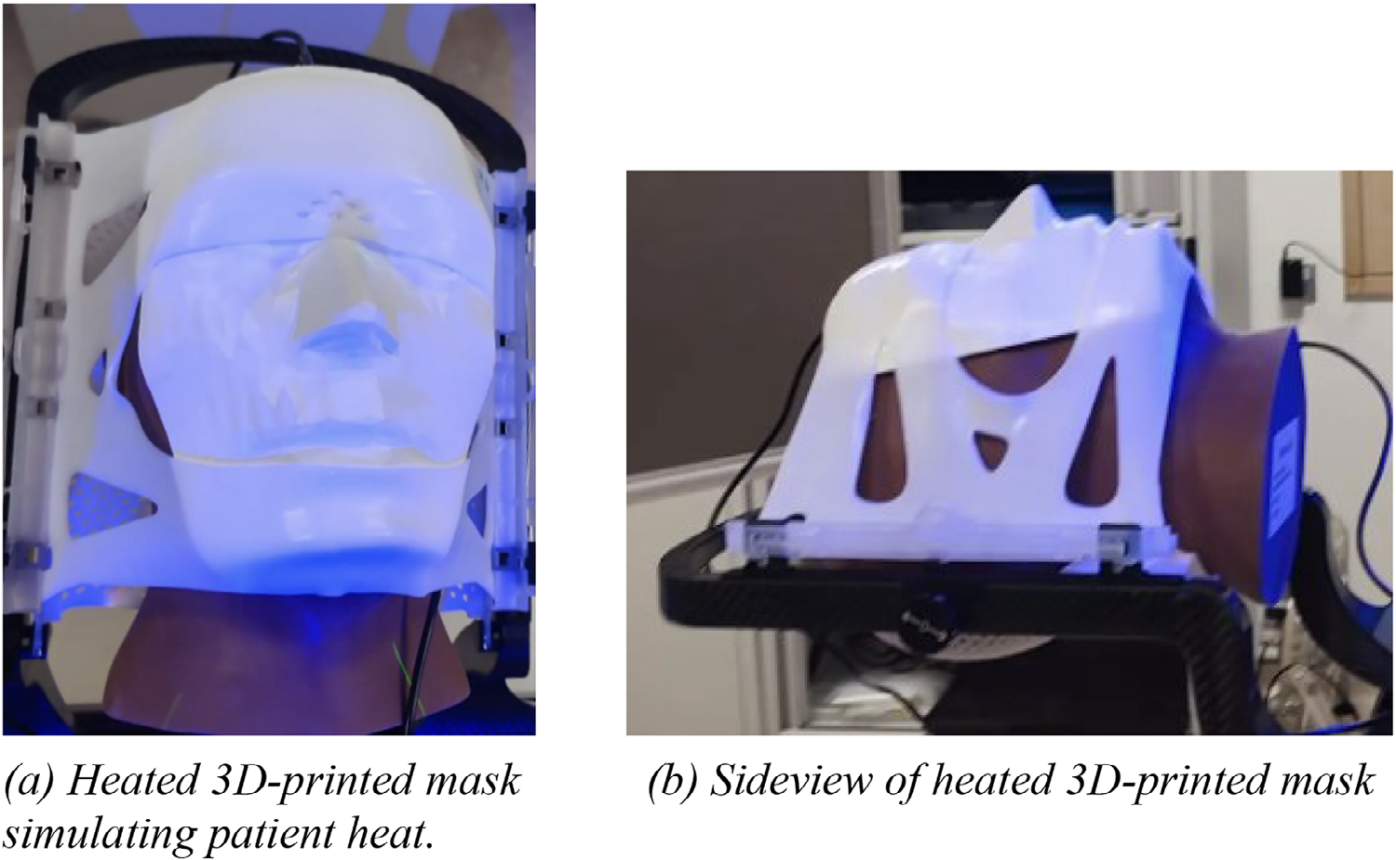
Heated 3D‐printed mask in front and side‐view.

This ROI covered the entire face, allowing precise monitoring of the phantom's movement, giving us insights into how well each mask restricted rotational displacements. Additionally, a heated 3D‐printed mask was placed on the phantom's face (Figure 3) to simulate the heat emitted by a patient's skin. Heat pads attached underneath the mask provided this heat, which was detected by ExacTrac's surface‐guided tracking system. ExacTrac combines both optical and heat detection to track any movements precisely.

To ensure consistency, the surface tracking region was manually defined over the same anatomical landmarks for all three masks. Although the Close Mask V1 and V2 contain hard plastic components that partially cover the surface, we verified that the ExacTrac Dynamic (ETD) thermal tracking still detected movement effectively without signal loss. The Open Mask, having no plastic obstruction, provided a larger uninterrupted surface tracking area. While this difference may introduce slight variability in tracking sensitivity, the relative differences between masks remain valid since all were evaluated under the same measurement conditions.

### 2.3 Final positioning and weight application

**FIGURE 4:**
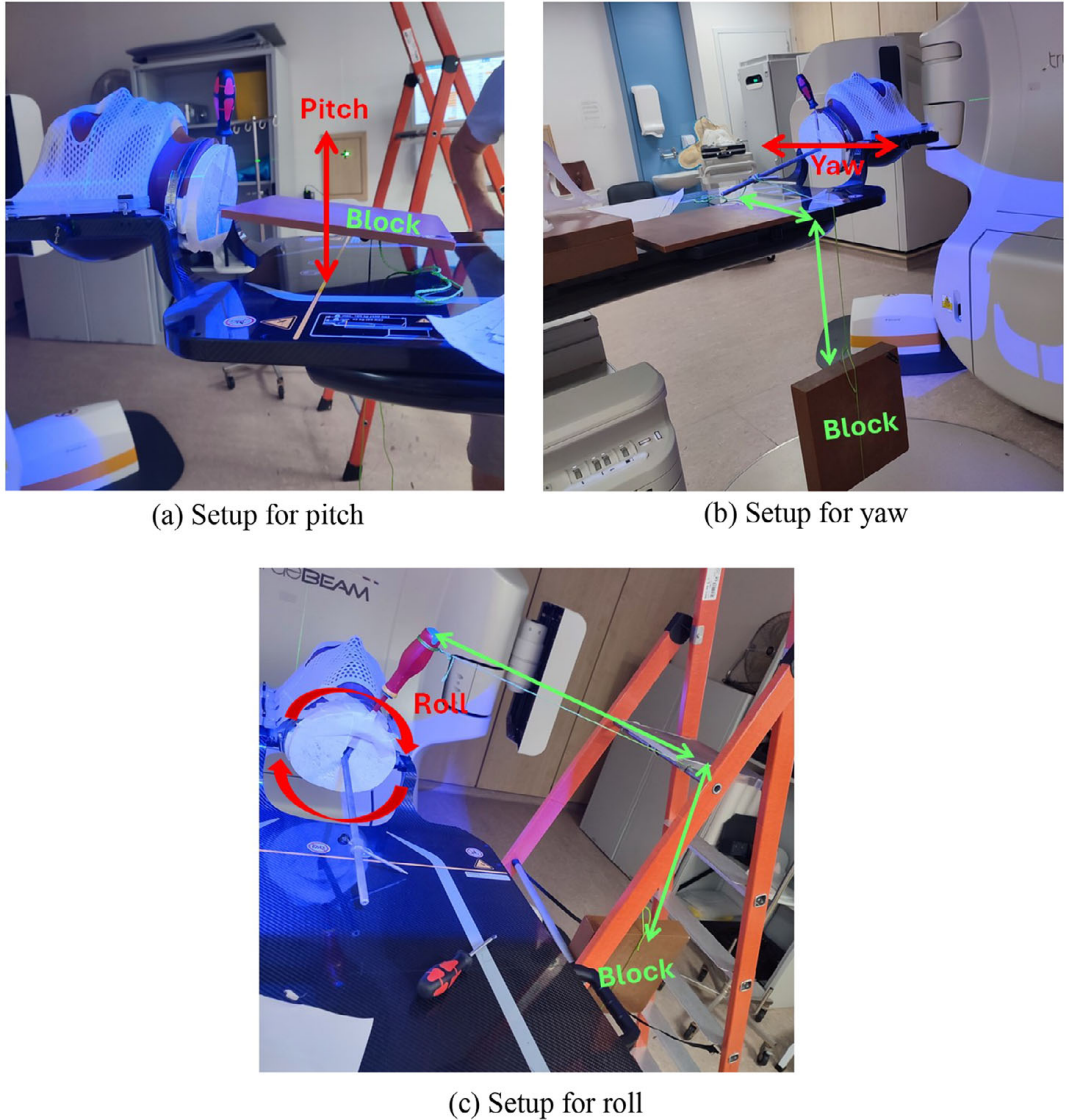
Head phantom setups for different movement simulations: (a) pitch, (b) yaw, and (c) roll.

The head phantom was secured with the selected mask and aligned using the linac's robotic couch. X‐ray images were taken and compared with Digitally Reconstructed Radiographs (DRRs) to verify alignment within 0.1 mm accuracy using the ExacTrac X‐ray 6D system. After each set of weights was removed, the phantom usually returned to its original position on its own. However, on a few occasions, it needed to be realigned using the same system to ensure it was back to its starting point. This step helped keep the starting conditions consistent for every test and reduced any uncertainties from repositioning. The fact that the phantom mostly returned to its original position on its own also confirmed that the displacements we observed were temporary and resolved once the weights were removed. Weights, which were QA water‐equivalent blocks, were attached to either the rod or the neck clamp, depending on the type of movement being induced. Specific setups were used for each movement direction:

- **For pitch movements**, the weight was placed on top of the long rod, which caused the head to tilt forward or backward, simulating pitch motion (Figure 4a).
- **For yaw movements**, a string was attached to the end of the central rod, and the weight was fastened to the other end of the string. This setup caused the head to rotate in the yaw direction (side‐to‐side) (Figure 4b).
- **For roll movements**, a screwdriver was placed into the 3D‐printed neck clamp, and a string was tied to it, pulling the weight in a way that caused the head to rotate left or right (see Figure 4c).

Each weight increment was followed by the measurement of rotational displacement. Measurements were taken using ExacTrac's surface‐guided thermal and optical tracking features, which detect movements with a resolution of 0.1°. This resolution introduces a rounding uncertainty, as displacements between 0.05° and 0.14° are all recorded as 0.1°. To mitigate the impact of this uncertainty, 4 measurements were taken for each movement, and variability across repetitions was accounted for in the analysis.

### 2.4 Force application and movement monitoring

To evaluate how well each mask restricted head movement, we applied incremental weights to the central rod attached to the head phantom. The weights were placed in specific positions to create movement in one of the three axes—pitch, yaw, or roll—similar to the rotational head movements patients can experience during Stereotactic Radiosurgery (SRS). The goal was to mimic movements typically seen in clinical settings, where rotational errors are usually within 0° to 1°.

Although calculating exact force values wasn't our primary focus, we concentrated on measuring the resulting displacements using the ExacTrac system. While the position of the weight can affect torque, in this study, the emphasis was on tracking the movement that occurred, rather than calculating precise forces or torque.

We started with a 656 g QA water block, which equates to 6.44N, as it was the smallest block available. After each additional 656 g weight was added, we carefully measured the rotational displacement in the pitch, yaw, and roll directions. Our objective was to keep these movements within the clinical threshold of less than 1°, essentially quantify rotational displacement under controlled forces and evaluate the effectiveness of different mask types in restricting movement. ExacTrac confirmed that the movements primarily occurred in the desired axis (e.g., pitch) while minimizing any unintended cross‐axis movements (e.g., yaw or roll during a pitch test).

### 2.5 Statistical analysis

To evaluate the efficacy of each mask in restricting movement, we conducted statistical analysis using Python 3.11 in the Spyder Integrated Development Environment (IDE). The analysis was performed with the SciPy library, specifically using the **ttest_rel** function to carry out paired *t*‐tests.

Paired *t*‐tests were conducted to compare the performance of each mask across the pitch, yaw, and roll directions. A significance level of *p* < 0.05 was set for this analysis. This threshold allowed us to determine whether there were statistically significant differences between the mask performances in minimizing head movements. The statistical analysis provided a clear comparison of the masks' stabilizing effects across all three axes of movement.

## 3 RESULTS AND ANALYSIS

The mean displacements and standard errors for each axis and mask type are shown in Table 1.

**TABLE 1:** Mean displacement and standard error for each mask type in pitch, yaw, and roll directions.

Mask type	Pitch (°)	Yaw (°)	Roll (°)
Close Mask V1	0.44 ± 0.13	0.28 ± 0.07	0.26 ± 0.07
Close Mask V2	0.20 ± 0.04	0.09 ± 0.02	0.16 ± 0.04
Open Mask	0.14 ± 0.03	0.11 ± 0.02	0.16 ± 0.03

The Open Mask showed the smallest mean displacement in pitch and roll, while the Close Mask V2 had the smallest mean displacement in yaw. The Close Mask V1 consistently showed higher displacements and greater variability, particularly in the pitch and roll directions.

**FIGURE 5:**
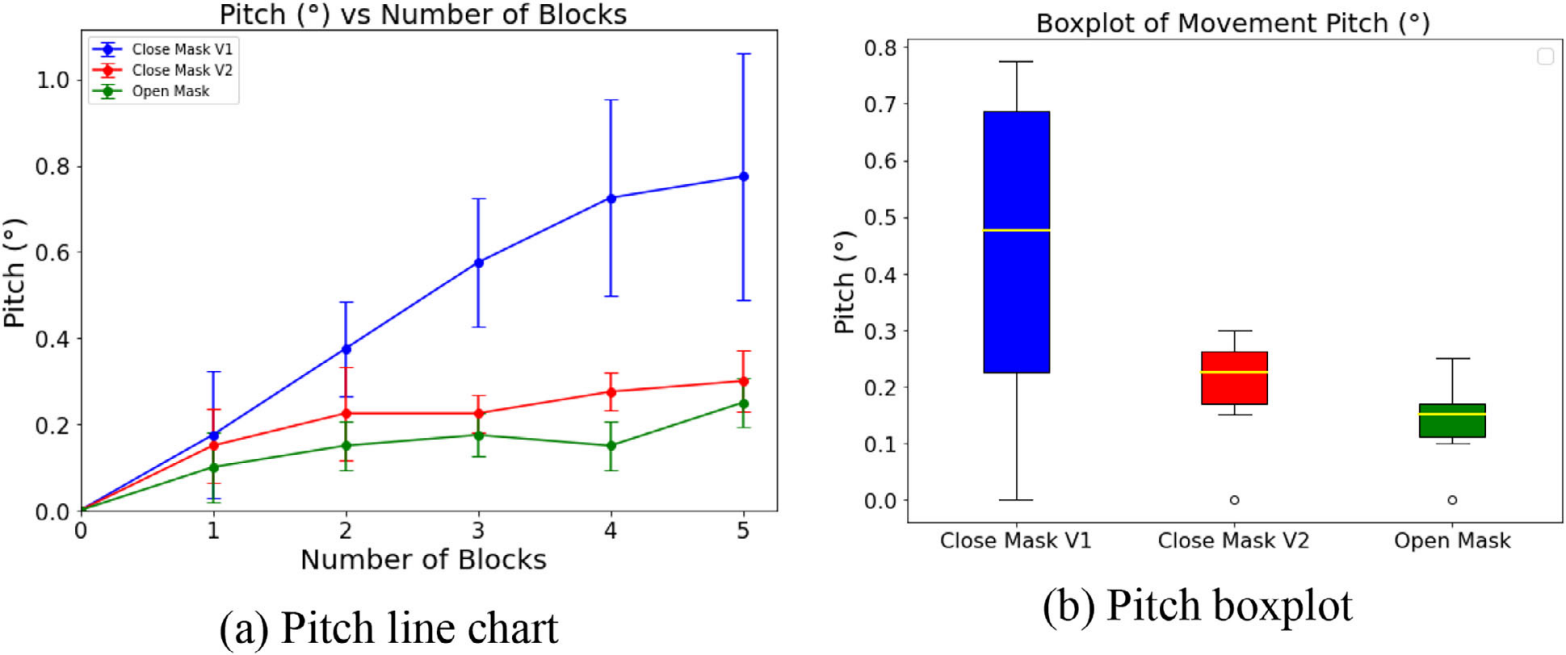
Pitch movement analysis for each mask type. (a) Line chart showing the relationship between the number of incremental weight blocks and pitch deviation (°). Each line corresponds to a mask type, with error bars indicating variability. (b) Boxplot showing the distribution of pitch deviations for different weight increments, with interquartile ranges and median values.

**FIGURE 6:**
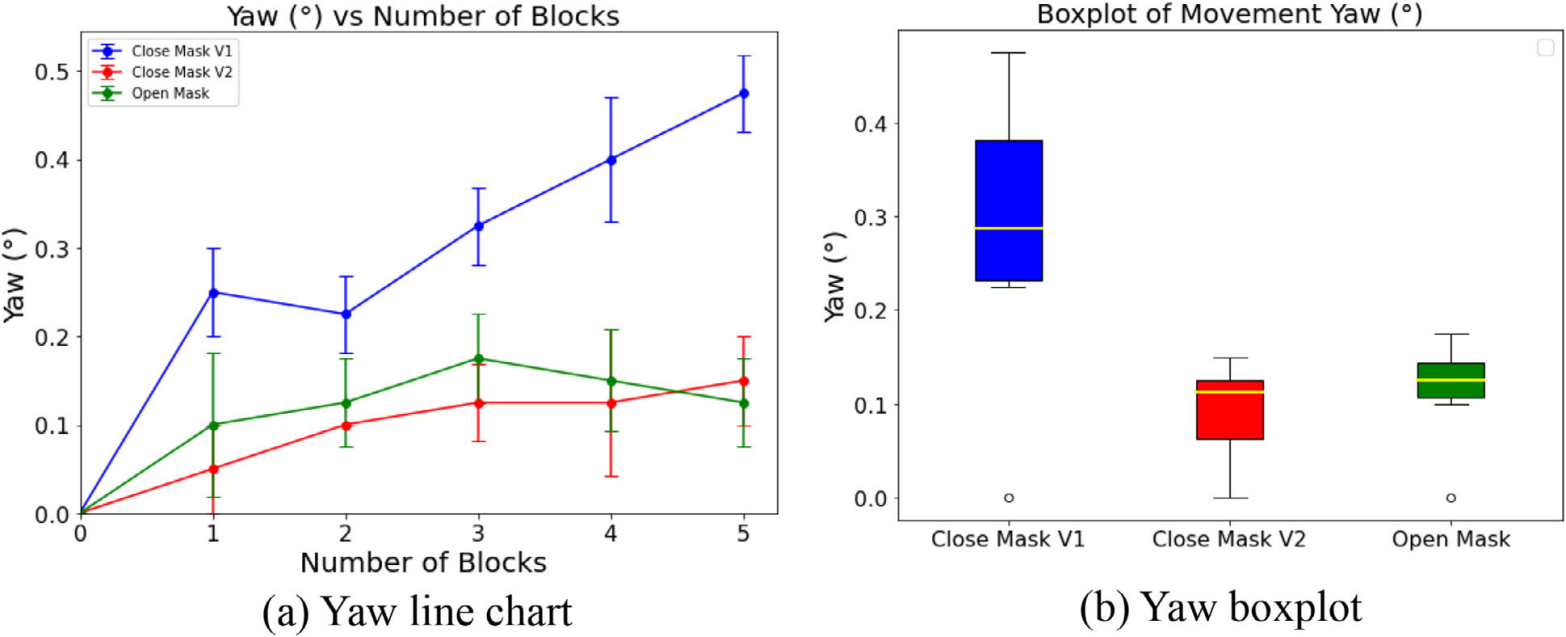
Yaw movement analysis for each mask type. (a) Line chart showing yaw deviation (°) versus the number of weight blocks. (b) Boxplot showing the range and median of yaw deviations for each weight increment.

**FIGURE 7:**
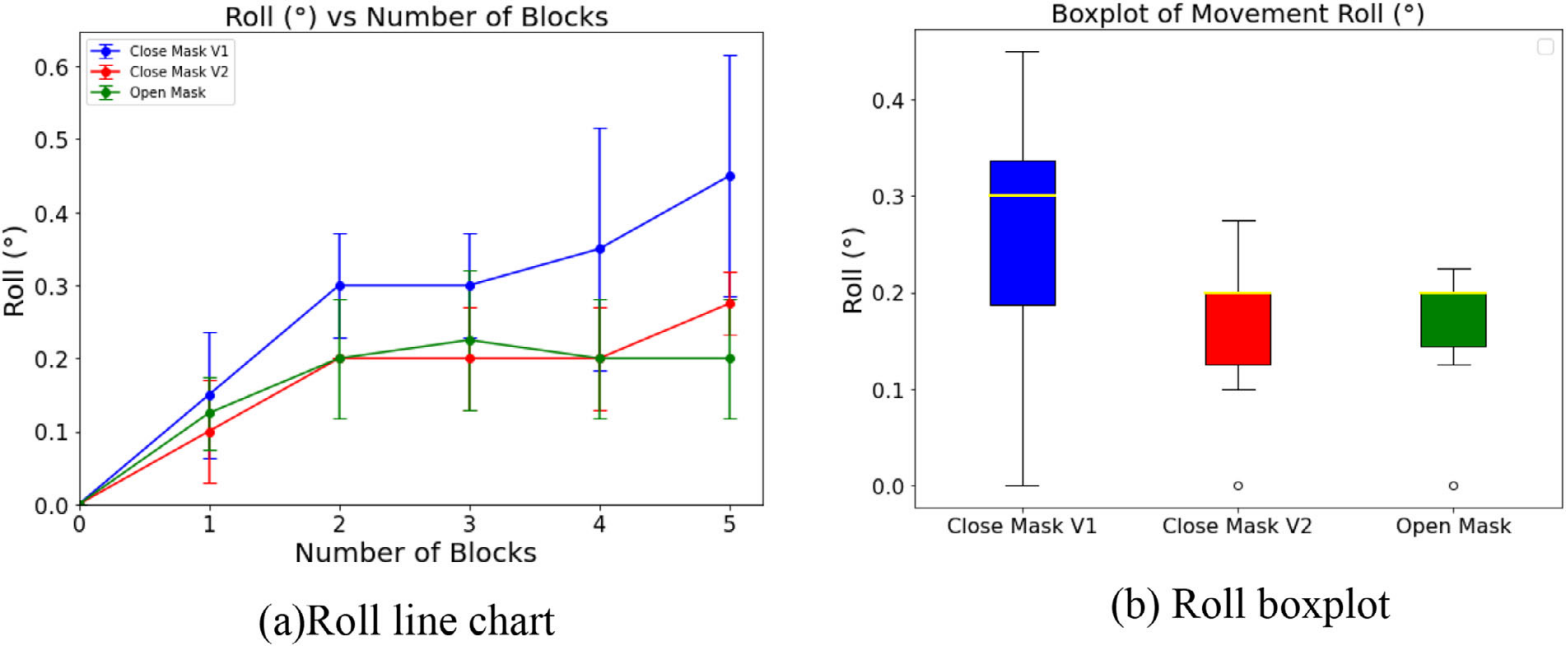
Roll movement analysis for each mask type. (a) Line chart showing roll deviation (°) versus the number of weight blocks. (b) Boxplot showing the distribution of roll deviations, highlighting variability across different mask types.

The line graphs (Figures 5, 6, 7a) clearly show how head movement changes as more weight blocks are added. Both the Open Mask and Stereotactic Mask effectively minimized movement, with differences in mean displacement values observed across axes.

In the pitch direction (Figure 5a), the Open Mask had the smallest movement, with a mean deviation of 0.14 ± 0.03°. Close Mask V2 was slightly higher at 0.20 ± 0.04°, while Close Mask V1 had the largest movement at 0.44 ± 0.13°.

For yaw movement (Figure 6a), the Open Mask and Close Mask V2 performed similarly, with mean deviations of 0.11 ± 0.02° and 0.09 ± 0.02°, respectively. In comparison, Close Mask V1 allowed more movement compared to the other two masks, with mean displacement differences of up to 0.28 ± 0.07°.

In the roll axis (Figure 7a), both the Open Mask and Close Mask V2 limited movement effectively, with mean deviations of 0.16 ± 0.03° and 0.16 ± 0.04°, respectively. Close Mask V1, however, showed larger variability and a higher deviation of 0.26 ± 0.07°.

In Figure 7b, the median (Q2) is equal to the third quartile (Q3) for Close Mask V2 and Open Mask, suggesting a skewed data distribution with most displacement values clustered toward the lower end. This pattern aligns with the limited rotational movement observed for these masks and was verified by reviewing the underlying data.

The boxplots (Figures 5, 6, 7b) highlight the range and variability of these movements. Close Mask V1 consistently had the widest ranges across all axes, showing less reliable immobilization. Close Mask V2 performed better, with narrower ranges, but the Open Mask consistently showed the tightest ranges, especially in pitch and yaw directions, indicating more stable and predictable performance.

Overall, these results show that the Open Mask provides the most consistent control across pitch, yaw, and roll movements. Close Mask V2 performs nearly as well, while Close Mask V1 lags behind, allowing more movement and variability. The focus here remains on comparing the masks' relative performance using weight increments as a practical measure to simulate head movement, rather than precise forces acting on the system.

### 3.1 Paired sample *t*‐test results

To assess the effectiveness of the three different thermoplastic masks in restricting head movement, we performed a paired samples t‐test for each mask pair. The tables below summarize the T‐statistics and *p*‐values for each movement type (pitch, yaw, and roll).

#### Close Mask V1 vs. Open Mask (Table 2)

**TABLE 2:** Paired t‐test results comparing Close Mask V1 and Open Mask.

Movement type	T‐statistic	*p*‐value
Pitch	3.09	0.0271
Yaw	3.37	0.0199
Roll	2.70	0.0429

The results show statistically significant differences in all movement directions between Close Mask V1 and the Open Mask. The Open Mask demonstrated superior performance in minimizing movement, particularly in the yaw axis, with a T‐statistic of 3.37 (p = 0.0199). Roll and pitch also showed significant improvements when using the Open Mask compared to Close Mask V1.

#### Close Mask V1 vs. Close Mask V2 (Table 3)

**TABLE 3:** Paired t‐test results comparing Close Mask V1 and Close Mask V2.

Movement type	T‐statistic	*p*‐value
Pitch	2.80	0.0380
Yaw	4.00	0.0103
Roll	3.66	0.0145

When comparing Close Mask V1 to Close Mask V2, the results again showed significant differences across all movement types. Close Mask V2 exhibited better performance, particularly in the yaw axis, with T‐statistics of 4.00 (*p* = 0.0103). This suggests Close Mask V2 offers better immobilization.

#### Close Mask V2 vs. Open Mask (Table 4)

**TABLE 4:** Paired t‐test results comparing Close Mask V2 and Open Mask.

Movement type	T‐statistic	*p*‐value
Pitch	3.50	0.0173
Yaw	−1.75	0.1412
Roll	0.28	0.7926

The comparison between Close Mask V2 and the Open Mask revealed a statistically significant difference in the pitch axis, with a T‐statistic of 3.50 (*p* = 0.0173). However, in the roll and yaw axes, the differences were not statistically significant, indicating comparable performance between the two masks in these directions.

From these results, the Open Mask and Close Mask V2 demonstrated comparable immobilization performance across most axes. In the yaw and roll directions, there were no statistically significant differences between the two masks, suggesting equivalent stabilization capabilities (yaw: *p* = 0.1412, roll: *p* = 0.7926). In the pitch direction, the Open Mask exhibited slightly better performance with a statistically significant difference (T = 3.50, *p* = 0.0173), though this difference was minor and clinically insignificant at less than 0.1°.

Close Mask V1, on the other hand, showed significantly more movement across all axes compared to both the Open Mask and Close Mask V2, making it less suitable for SRS. These findings reinforce the reliability of both the Open Mask and Close Mask V2 for ensuring accurate head immobilization during SRS, with the Open Mask offering comparable performance to Close Mask V2 in all but the pitch axis, where the difference remains clinically negligible.

## 4 DISCUSSION

This study evaluated the effectiveness of different mask types in reducing rotational head movement under controlled force application. While relevant for SRS applications, the study did not directly assess immobilization performance during clinical treatments. We found that the Cranial 4pi open‐face mask and the stereotactic mask (Close Mask V2) performed equally well in limiting rotational movements, especially in the pitch and yaw directions. The basic mask, on the other hand, showed more movement and is likely less suitable for precise treatments like SRS.

While we applied uniform weights to simulate force on the head phantom, it's important to point out that torque—not just force—determines how much rotation happens. Torque depends on both the force applied and the distance from the axis of rotation, as well as the angle of application. This may explain why we didn't see movements consistently increase with added weight, particularly for yaw and roll with the open‐face mask. Instead of focusing on exact force values, we measured movements relative to the number of weight blocks applied. This approach kept the focus on how the masks compared to each other rather than getting into precise calculations of force and torque.

One notable advantage of the open‐face mask is its ability to combine immobilization with greater patient comfort, an important factor in reducing patient anxiety and improving compliance during treatments. As highlighted in other studies, achieving sub‐millimetre and sub‐degree accuracy is critical for SRS, and maintaining patient comfort through more breathable masks can play a role in reducing involuntary movement during long sessions.^11^, ^12^ This matters in SRS because even slight misalignments can lead to unintended radiation exposure to healthy tissue or underdosing of the target area.

This study has several limitations. While using a head phantom allowed for controlled and repeatable testing, it does not fully replicate the anatomical variability found in patients. Factors such as jawbone structure and skin elasticity, which can affect mask fit, were not accounted for in this setup.^13^, ^14^ Additionally, only a single unit of each mask type was tested, meaning potential manufacturing inconsistencies and material variations were not assessed. We assumed minimal variability between different mask units of the same type, due to the standardized manufacturing process of Brainlab masks. Future studies should incorporate multiple mask sets to evaluate batch‐to‐batch variability and the impact of repeated clinical use.

Another limitation is the difference in surface tracking conditions across mask types. Although we defined the ROI consistently, the hard plastic components of Close Mask V1 and V2 may have influenced ExacTrac's optical/thermal tracking sensitivity. Future research could integrate X‐ray tracking to validate surface‐tracking results and eliminate any potential bias introduced by mask material differences.

The ExacTrac Dynamic (ETD) system has a resolution of 0.1°, which introduces an inherent uncertainty in displacement measurements. While we performed four repeated measurements per movement type to mitigate this, the potential uncertainty propagation was not explicitly accounted for in the statistical analysis. Future studies should incorporate uncertainty propagation in statistical significance testing to refine confidence intervals and improve measurement accuracy.

Finally, while our findings provide insight into mask performance under controlled conditions, real‐world patient data would further validate these results. Future work should assess mask effectiveness in a clinical setting over extended use to ensure long‐term immobilization stability.

It's also important to note that our results are specific to the solid two‐piece masks tested here. These findings may not apply to other designs, like single‐piece or perforated open masks. More research is needed to explore how other mask types perform under similar conditions.

In this study, we used the ExacTrac system's surface‐guided tracking feature to measure rotational movements (pitch, yaw, and roll) under controlled force application. While ExacTrac includes an X‐ray correction function, we only used it to realign the phantom when it did not return to its initial position after weight removal. Our choice to use surface‐tracking was driven by convenience—allowing efficient, repeated measurements without requiring room re‐entry—rather than an assessment of its accuracy as a tracking system. Future studies could compare surface‐tracking with X‐ray tracking to determine their relative accuracy and limitations.

Due to the 0.1° measurement resolution of the ExacTrac system, statistical significance should be interpreted with caution, particularly for displacement differences close to this threshold. While paired *t*‐tests indicate differences between mask types, the inherent resolution limit introduces uncertainty in detecting small variations. Future research should incorporate uncertainty propagation in statistical analysis to refine confidence intervals and improve result reliability.

Overall, both the open‐face mask and the stereotactic mask performed very well in this study and are reliable options for immobilization in SRS. The open‐face mask, in particular, offers the added benefit of improved patient comfort without compromising precision, making it a reliable option for high‐accuracy treatments.^15^

## 5 CONCLUSION

This study looked at how well three Brainlab thermoplastic masks could keep the head still during Stereotactic Radiosurgery (SRS). The results showed that the Cranial 4pi open‐face mask and the stereotactic mask (Close Mask V2) performed equally well in most cases. Both masks were able to minimize movement in yaw and roll directions, with no major differences (*p* = 0.1412 and *p* = 0.7926). In the pitch direction, the open‐face mask was slightly better, but the difference was very small—less than 0.1° (T‐statistic = 3.50, *p* = 0.0173). On the other hand, the basic mask allowed much more movement in all directions, making it less suitable for precise treatments like SRS.

These results show that the open‐face and stereotactic masks are equally good at keeping the head stable during SRS. This means both masks are reliable options for delivering accurate radiation treatment. The open‐face mask could also be chosen for its potential comfort benefits, depending on the patient's needs.

## AUTHOR CONTRIBUTIONS

**I**ris Pasion Apale: Designed the study, analyzed the data, and wrote the manuscript. Adam Agnew: Designed the study, contributed to the experimental setup and data collection, and reviewed the manuscript. Daniel Foley: Contributed to the experimental setup and data collection, and reviewed the manuscript.

## CONFLICT OF INTEREST STATEMENT

The authors declare no conflicts of interest.

## ETHIC STATEMENT

This study was conducted in accordance with the ethical standards of the relevant institutional review board or ethics committee.

## Data Availability

The data supporting the findings of this study are available upon reasonable request from the corresponding author.

## References

[1] Seung S , Larson D , Galvin J , et al. American College of radiology (ACR) and american society for radiation oncology (ASTRO) practice guideline for the performance of stereotactic radiosurgery (SRS). Am J Clin Oncol. 2013;36(3):310‐315. doi:10.1097/coc.0b013e31826e053d 23681017 PMC4285440

[2] Ryu S , Yin F , Rock J , et al. Image‐guided and intensity‐modulated radiosurgery for patients with spinal metastasis. Cancer. 2003;97(8):2013‐2018. doi:10.1002/cncr.11296 12673732

[3] Nixon J , Cartmill B , Turner J , et al. Exploring the prevalence and experience of mask anxiety for the person with head and neck cancer undergoing radiotherapy. J Med Radiat Sci. 2018;65(4):282‐290. doi:10.1002/jmrs.308 30378282 PMC6275267

[4] Linthout N , Verellen D , Tournel K , Storme G . Six dimensional analysis with daily stereoscopic x‐ray imaging of intrafraction patient motion in head and neck treatments using five points fixation masks. Med Phys. 2006;33(2):504‐513. doi:10.1118/1.2165417 16532958

[5] Ohira S , Kanayama N , Komiyama R , et al. Intrafractional patient setup error during fractionated intracranial stereotactic irradiation treatment of patients wearing medical masks: comparison with and without bite block during COVID‐19 pandemic. J Radiat Res. 2020;62(1):163‐171. doi:10.1093/jrr/rraa101 PMC7717301 33392618

[6] Mangesius J , Seppi T , Weigel R , et al. Intrafractional 6D head movement increases with time of mask fixation during stereotactic intracranial RT sessions. Radiat Oncol. 2019;14(1). doi:10.1186/s13014-019-1425-7 PMC6921566 31852497

[7] Weltens C , Kesteloot K , Vandevelde G , Bogaert WVD . Comparison of plastic and Orfit® masks for patient head fixation during radiotherapy: precision and costs. Int J Radiat Oncol Biol Phys. 1995;33(2):499‐507. doi:10.1016/0360-3016(95)00178-2 7673040

[8] Contesini M , Guberti M , Saccani R , et al. Setup errors in patients with head‐neck cancer (HNC), treated using the intensity modulated radiation therapy (IMRT) technique: how it influences the customised immobilisation systems, patient's pain and anxiety. Radiat Oncol. 2017;12(1). doi:10.1186/s13014-017-0807-y PMC5408424 28449698

[9] Minniti G , Clarke E , Lanzetta G , et al. Stereotactic radiosurgery for brain metastases: analysis of outcome and risk of brain radionecrosis. Radiat Oncol. 2011;6(1). doi:10.1186/1748-717x-6-48 PMC3108308 21575163

[10] Babic S , Lee YK , Ruschin M , et al. To frame or not to frame? Cone‐beam CT‐based analysis of head immobilization devices specific to linac‐based stereotactic radiosurgery and radiotherapy. J Appl Clin Med Phys. 2018;19(2):111‐120. doi:10.1002/acm2.12251 29363282 PMC5849846

[11] Li G , Lovelock D , Mechalakos J , et al. Migration from full‐head mask to “open‐face” mask for immobilization of patients with head and neck cancer. J Appl Clin Med Phys. 2013;14(5):243‐254. doi:10.1120/jacmp.v14i5.4400 24036878 PMC5714571

[12] Zhou S , Li J , Zhu X , et al. Initial clinical experience of surface guided stereotactic radiation therapy with open‐face mask immobilization for improving setup accuracy: a retrospective study. Radiat Oncol. 2022;17(1). doi:10.1186/s13014-022-02077-4 PMC9167505 35659685

[13] Sweeney R , Bale R , Vogele M , et al. Repositioning accuracy: comparison of a noninvasive head holder with thermoplastic mask for fractionated radiotherapy and a case report. Int J Radiat Oncol Biol Phys. 1998;41(2):475‐483. doi:10.1016/S0360-3016(98)00064-9 9607367

[14] Bale R , Vogele M , Freysinger W , et al. Minimally invasive head holder to improve the performance of frameless stereotactic surgery. Laryngoscope. 1997;107(3):373‐377. doi:10.1097/000055 9121316

[15] Keane M , Weitkamp N , Madani I , et al. Randomized self‐controlled study comparing open‐face vs. closed immobilization masks in fractionated cranial radiotherapy. Radiotherapy and oncology. 2024:110314‐110324. doi:10.1016/j.radonc.2024.110314 38677329

